# Dynamical magnetic behavior of anisotropic spinel-structured ferrite for GHz technologies

**DOI:** 10.1038/s41598-020-79768-z

**Published:** 2021-01-12

**Authors:** Yukiko Yasukawa, Kouhei Nozawa, Taneli Tiittanen, Maarit Karppinen, Johan Lindén, Sagar E. Shirsath, Shin Yabukami

**Affiliations:** 1 Department of Electrical and Electronic Engineering, Faculty of Engineering, Chiba Institute of Technology, 2-17-1 Tsudanuma, Narashino, Chiba 275-0016 Japan; 2 Graduate School of Engineering, Tohoku University, Building No. 2, 6-6-05 Aoba Aza Aramaki, Aoba, Sendai, Miyagi 980-8579 Japan; 3 Department of Chemistry and Materials Science, Aalto University, 00076 Espoo, Finland; 4 Physics/Faculty of Science and Engineering, Åbo Akademi, Porthansg. 3, 20500 Turku, Finland; 5 School of Materials Science and Engineering, University of New South Wales, Sydney, NSW 2052 Australia

**Keywords:** Engineering, Materials science

## Abstract

We have fabricated a high quality magnetic Ni_0.5_Zn_0.5_Fe_2_O_4_ ferrite powder/polymer composite sheet consisting of common and environmentally friendly elements only. The sheet was then tested for its dynamic permeability by irradiating with electromagnetic waves with frequencies up to 50 GHz. Two different originally developed methods were used for the high-frequency permeability measurements, a short-circuited microstrip line method and a microstrip line-probe method. It is challenging to measure the dynamic permeability of magnetic thin films/sheets beyond 10 GHz because of the low response signal from these materials. However, the two methods produced essentially equivalent results. In the frequency dependent permeability profile, the maximum position of the profile, $\mu ^{\prime \prime }_{max}$, shifted towards higher frequencies upon increasing an applied (strong) static external magnetic field, $H_{dc}$. A linear relationship between $\mu^ {\prime \prime }_{max}$ and $H_{dc}$ for the entire range of $H_{dc}$ was observed even at small $H_{dc}$. In general, the spinel-structured Ni-based ferrites exhibit low magnetic anisotropy, but the present sample showed a uniaxial-anisotropic behavior in the parallel direction of the sheet. Our Ni_0.5_Zn_0.5_Fe_2_O_4_ powder/polymer composite sheet thus exhibits high performance at GHz frequencies, and should be applicable e.g. as an anisotropic electromagnetic wave-interference material.

## Introduction

Current telecommunication systems and electronic devices with super-high functionalities are operated by GHz-frequency electromagnetic waves. Consequently, magnetic materials working in the high GHz-frequency region are indispensable for e.g. sensing and spintronic torque-oscillation devices, electromagnetic wave absorbers, and magnetic sheets for radio-frequency identification (RFID). For the practical use of these magnetic materials, dynamic permeability behaviors under the irradiation of high-frequency electromagnetic waves is a key issue, because permeability determines the applicable frequency bands of magnetic-based devices. For instance, the resonance frequency ($f_{r}$) should be in the MHz region for a magnetic-based electromagnetic wave shield, which is applied to wireless-power transmission circuits^1^. On the other hand, magnetic materials exhibiting $f_{r}$ values in the GHz region can be used as electromagnetic wave absorbers inside mobile phones.

Typically, the dynamic permeability of the GHz region is evaluated based on reflection or transmission methods. In these methods, microstrip line (MSL), coaxial and coplanar waveguides are commonly used. The measurement results obtained from experiments are usually analyzed by the lumped-element approximation^2,3^, or the distributed-element approximation^4,5^. Moreover, some groups measure the high-frequency permeability using commercialized-product devices, while others develop new devices aiming at more accurate measurements/analyses for the higher frequencies of the “5G era”. Thus, various combinations of experimental devices, measurement techniques, and analysis methods have been employed to evaluate the dynamic permeability, such that the measurement/analysis procedures are often strongly specific to the different research groups^6−11^.

Ferrites are promising materials for high-frequency devices^12^ because of their ferromagnetic resonance (FMR) in the MHz- to GHz-frequency region. High resistivity but low conduction losses in the high-frequency region is another prominent advantage of ferrites. Magnetoplumbite-structured ferrites with a chemical formula of *A*Fe_12_O_19_ (*A* = alkali or alkaline-earth metal) have been widely studied, as $f_{r}$ is expected to reach as high as ~ 50 GHz for these materials^8,13−15^. For example, in a Sm-doped high purity single-crystal SrFe_12_O_19_ $f_{r}$ was found to be 53 GHz^16^. However, keeping an eye on the recent high-frequency technologies, the electromagnetic wave interference (EMI) may become a serious problem. In this regard, the magnetoplumbite-structured *A*Fe_12_O_19_ ferrites^17^, spinel-structured Ni_1-x_Zn_x_Fe_2_O_4_ ferrites, and soft magnetic alloy materials mixed with polymers^18^ are expected to be attractive materials, especially in the field of EMI at GHz bands.

In this study, we evaluate the dynamic permeability behavior of ferrites under the irradiation of an electromagnetic wave of GHz frequency through two different originally developed measurement techniques, (i) short-circuited MSL^10,15,19^, and (ii) MSL-probe^11,20^ methods. Our aim is to provide a comprehensive understanding of the dynamical magnetic behaviors of ferrites in the GHz region. For both measurement methods, the same sample, i.e., Ni_0.5_Zn_0.5_Fe_2_O_4_ powder/polymer composite sheet from the same sample batch, was used. We used polyvinyl alcohol (PVA) as a polymer source. It should be noted that the spinel-structured Ni_0.5_Zn_0.5_Fe_2_O_4_ is widely commercialized because of its simpler crystal structure compared to the *A*Fe_12_O_19_ ferrites with the magnetoplumbite structure. Therefore, it is an optimal material for the present study.

## Results and discussion

### Verification of the quality of the Ni_0.5_Zn_0.5_Fe_2_O_4_ powder

**Figure 1:**
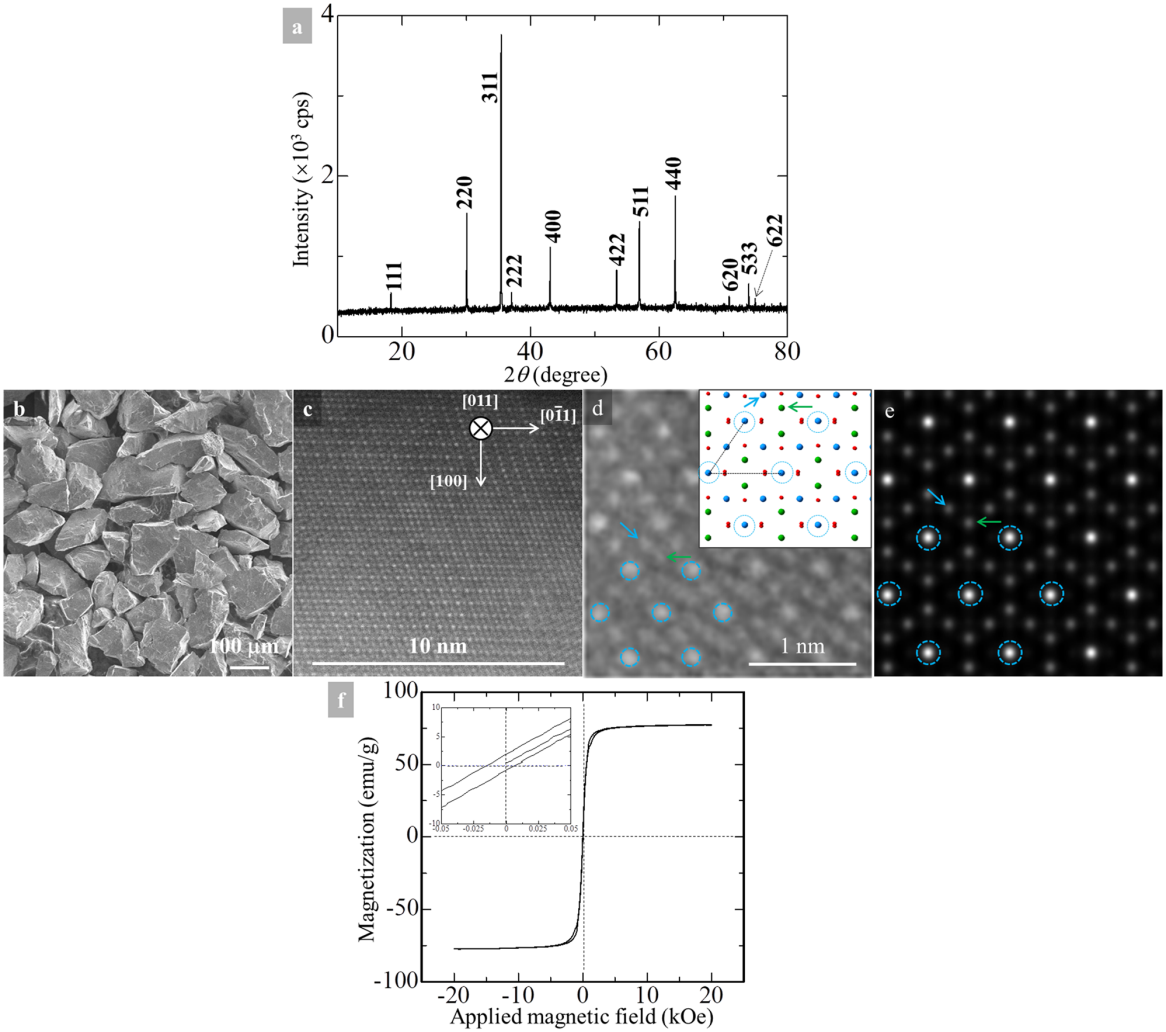
Fundamental characteristics of the Ni_0.5_Zn_0.5_Fe_2_O_4_ ceramic powder synthesized by a solid-state reaction method after the final heat-treatment. (**a**) XRD pattern of polycrystalline Ni_0.5_Zn_0.5_Fe_2_O_4_, (**b**) FE-SEM and (**c**) HAADF images of Ni_0.5_Zn_0.5_Fe_2_O_4_. The HAADF image was taken with an incident electron beam from [011]. (**d**) is an enlarged image of (**c**) with simulated atomic positions (insertion). The simulation was based on a reference^21^. Symbols used are as follows; O (red circles), *A*-site cations at tetrahedrons (green circles), *B*-site cations at octahedrons (blue-dashed circles), another *B*-site cation at octahedron (blue arrow). (**e**) Simulated diffraction patterns of a spinel-structured ferrite with a thickness of ~ 4.2 nm. (**f**) Magnetic hysteresis loop of Ni_0.5_Zn_0.5_Fe_2_O_4_ ceramic powder measured at room temperature. Inset shows enlarged the vicinity of coercivity.

The high sample quality was an essential requirement for the present study. Hence we started the study by confirming the phase purity and the fundamental structural and physical properties of the synthesized Ni_0.5_Zn_0.5_Fe_2_O_4_ ceramic powder. First of all, the XRD pattern shown in Fig. 1a confirms the cubic spinel structure (lattice parameter determined at 8.398 Å in excellent agreement with the value of 8.383 Å given in the JCPDS card) for the sample without any impurity phase peaks. Moreover, the half-width-at-half-maximum (HWHM) value of 0.05° for the strongest (311) peak indicates excellent crystallinity.

Further evidence of the high sample quality of our Ni_0.5_Zn_0.5_Fe_2_O_4_ ceramic powder was obtained from the microstructural study. From the FE-SEM image (Fig. 1b) it could be seen that the powder consists of crystallites larger than 100 μm. The local-crystal structure observation by high-angle annular dark-field (HAADF) with an incident-electron beam from [011] revealed well-ordered arrangements of atoms (Fig. 1c). Moreover, we confirmed the Ni, Zn, Fe, and O signals from the TEM-EDS analysis. Finally, Fig. 1d displays an enlarged image of Fig. 1c, and its inset shows the simulated atomic positions of the spinel-structured ferrite. For this simulation, the atomic positions were taken from a literature^21^. In the simulated image, oxygen atoms are drawn in red, *A*-site cations in tetrahedra in green, and *B*-site cations in octahedra in blue. There are two distinct *B*-site cations in the simulated image, marked by blue circles and a blue arrow. It is noteworthy to point out that these two types of *B*-site cations can be recognized in Fig. 1d. The simulated atomic positions and the atomic configurations are consistent with the interpretation of Fig. 1d. In Fig. 1e we display a simulated diffraction pattern for a spinel-structured ferrite with a thickness of 4.2 nm. By comparing the observed (Fig. 1d) and simulated (Fig. 1e) images, the high/low-color contrasts of both the images are consistent with the following interpretation: *B*-site cations marked by blue circles exhibit higher contrast, while the position exhibiting indistinct contrast is due to atoms indicated by a blue arrow. Furthermore, the *A*-site cation indicated by a green arrow exhibits low contrast in Fig. 1d, which agrees with the simulated result (Fig. 1e).

We also confirmed the magnetic property of our Ni_0.5_Zn_0.5_Fe_2_O_4_ ceramic powder; from Fig. 1f it shows the typical soft magnetic behavior with a low coercivity ($H_{c}$) value of 13 Oe, and saturation-magnetization ($M_{s}$) of 78 emu/g, in agreement with the values reported by other groups^22,23^. Mössbauer spectroscopy enabled us to simultaneously determine the oxidation state, chemical surrounding and magnetic structure; we performed Mössbauer measurements on our Ni_0.5_Zn_0.5_Fe_2_O_4_ ceramic powder at room temperature without applying an external magnetic field. The spectrum consisted of several magnetically broadened components and one paramagnetic component. The paramagnetic component was assigned to a Fe atom surrounded only by Zn atoms, thereby the local magnetic coupling of this Fe species was broken. The isomer shift values indicated that all the Fe atoms in the Ni_0.5_Zn_0.5_Fe_2_O_4_ ceramic powder are trivalent (${\text{Fe}}^{3 + }$) in a high-spin state without any traces of divalent Fe (${\text{Fe}}^{2 + }$). This confirms that the present sample is fully oxygenated, indicating that a high resistivity is expected.

### Static physical behavior of the Ni_0.5_Zn_0.5_Fe_2_O_4_ powder/polymer composite sheet

**Figure 2:**
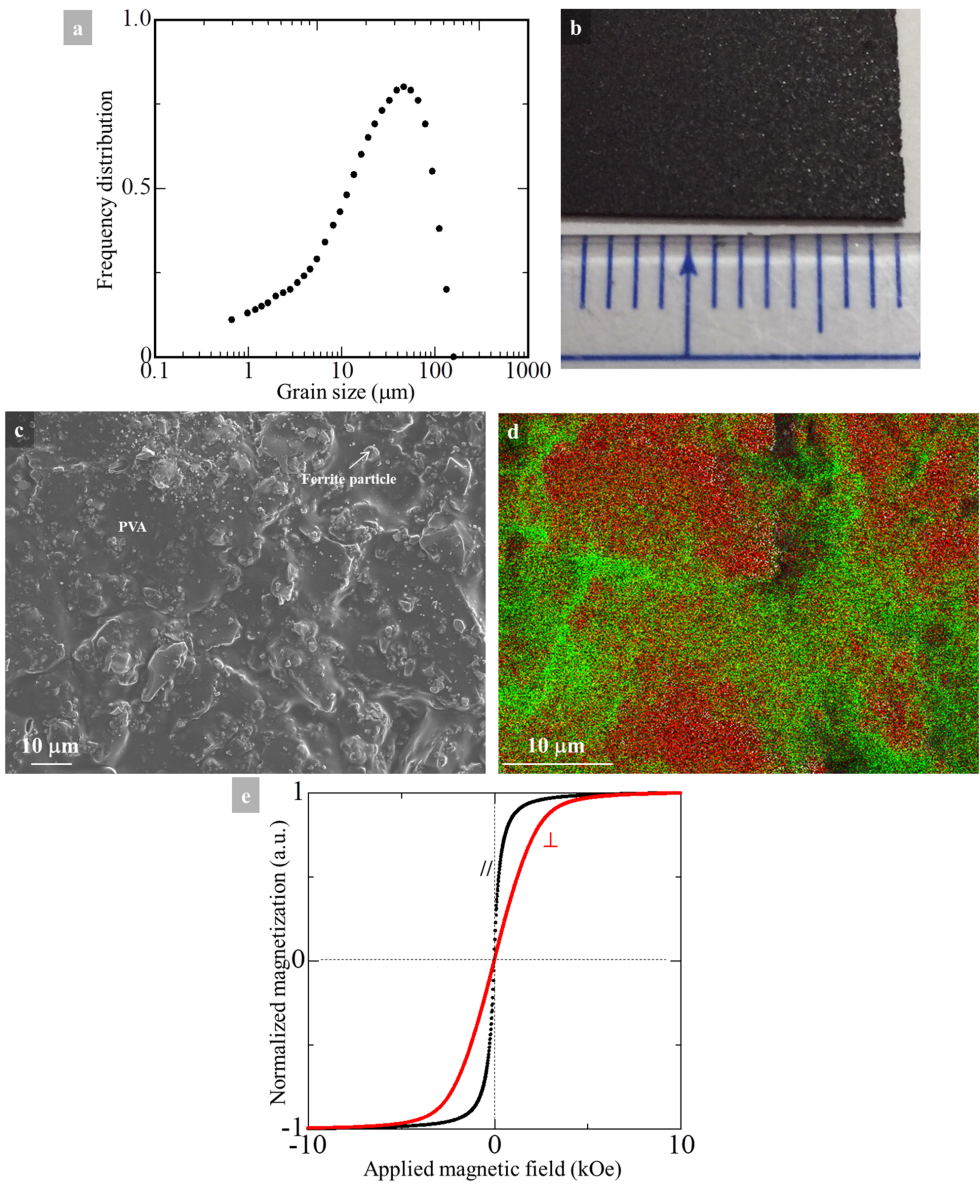
(**a**) Distribution of particle size of ceramic Ni_0.5_Zn_0.5_Fe_2_O_4_ powder for the preparation of Ni_0.5_Zn_0.5_Fe_2_O_4_ powder/polymer composites sheet. (**b**) The physical appearance of the sheet, and (**c**) the microstructures of the sheet taken by FE-SEM. The distribution of elements in the sheet is shown in (**d**). Fe is represented by red color, while green shows the C distribution. Magnetic hysteresis characteristics of the Ni_0.5_Zn_0.5_Fe_2_O_4_ powder/polymer composite sheet at room temperature are shown in (**e**). The external magnetic field is applied normal to the sheet surface (red), whereas the black loop is obtained when the external magnetic field is applied parallel to the surface of the sheet.

Prior to the preparation of sheet-shaped Ni_0.5_Zn_0.5_Fe_2_O_4_ powder/polymer composites, the Ni_0.5_Zn_0.5_Fe_2_O_4_ ceramic powder was crushed using a grinding machine to achieve homogeneous-sized particles leading to homogeneous physical properties. Afterward, the powder was classified based on the sizes of particles by the use of 180, 120, and 90 μm sieves, successively. The distribution of the particle size of the ceramic Ni_0.5_Zn_0.5_Fe_2_O_4_ powder after the classification is shown in Fig. 2a. The largest number of particles occurs at approximately 50 μm, while the median value of the particle size is 25 μm. By using homogeneous-sized Ni_0.5_Zn_0.5_Fe_2_O_4_ ceramic powder, we prepared a sheet-shaped Ni_0.5_Zn_0.5_Fe_2_O_4_ powder/polymer composite as described in the Method section. The appearance of the prepared sheet is shown in Fig. 2b. The average thickness of the sheet is 225 μm. The FE-SEM image shown in Fig. 2c reveals that Ni_0.5_Zn_0.5_Fe_2_O_4_ particles with various shapes are embedded in the polymer matrix. Also seen is that the sheet exhibits some surface roughness. The distribution of constituent elements of the sheet analyzed by EDS is in Fig. 2d; in the EDS mapping red corresponds to Fe, whereas C is denoted as green. It is inferred that the location of Fe corresponds to the existence of ferrite particles. In contrast, the signal from C shows the distribution of the polymer. In the present sheet, C is selectively detected at the boundary of the Ni_0.5_Zn_0.5_Fe_2_O_4_ particles, thereby each Ni_0.5_Zn_0.5_Fe_2_O_4_ particle is isolated by the polymer matrix. The surface resistivity of the Ni_0.5_Zn_0.5_Fe_2_O_4_-powder/polymer composite sheet measured under an application voltage of 10 V at room temperature is 1.09 × 10^9^ Ω, which is sufficiently high. Thus, the influence of eddy currents in the sheet can be ruled out when high frequency electromagnetic wave is irradiated on the sample. This is a great advantage for materials applied in high-frequency technologies.

The magnetic properties of the Ni_0.5_Zn_0.5_Fe_2_O_4_-powder/polymer composite sheet were measured by applying an external magnetic field parallel with (//) and perpendicular to (⊥) the surface of the sheet (Fig. 2e). Note that the magnetization value shown in Fig. 2e is normalized. The Ni_0.5_Zn_0.5_Fe_2_O_4_ ceramic powder exhibited soft magnetic behavior (see Fig. 1f), and the sheet basically repeated this behavior. Indeed, $H_{c}$ values for the Ni_0.5_Zn_0.5_Fe_2_O_4_-powder/polymer composite sheet are only 12 Oe for parallel, and 17 Oe for perpendicular configurations. There is a configuration dependence for the saturation-magnetic field in Fig. 2e, which suggests a presence of magnetic anisotropy, i.e., that the magnetic easy axis is along the parallel direction in the sheet. The distribution of each Ni_0.5_Zn_0.5_Fe_2_O_4_ particle is not artificially controlled inside the sheet (see Fig. 2c), so that the magnetic properties of the Ni_0.5_Zn_0.5_Fe_2_O_4_ particles dispersed in the sheet should vary from particle to particle. The different magnetic behaviors seen in Fig. 2e between the parallel and perpendicular configurations could be induced by a shape anisotropy, which is generated during the sheet preparation by the Doctor-Blade method (see Method section). As a consequence, we infer that the present Ni_0.5_Zn_0.5_Fe_2_O_4_-powder/polymer composite sheet would be the parallel (in-plane) magnetic anisotropy.

### Dynamic physical behavior of the Ni_0.5_Zn_0.5_Fe_2_O_4_ powder/polymer composite sheet

**Figure 3:**
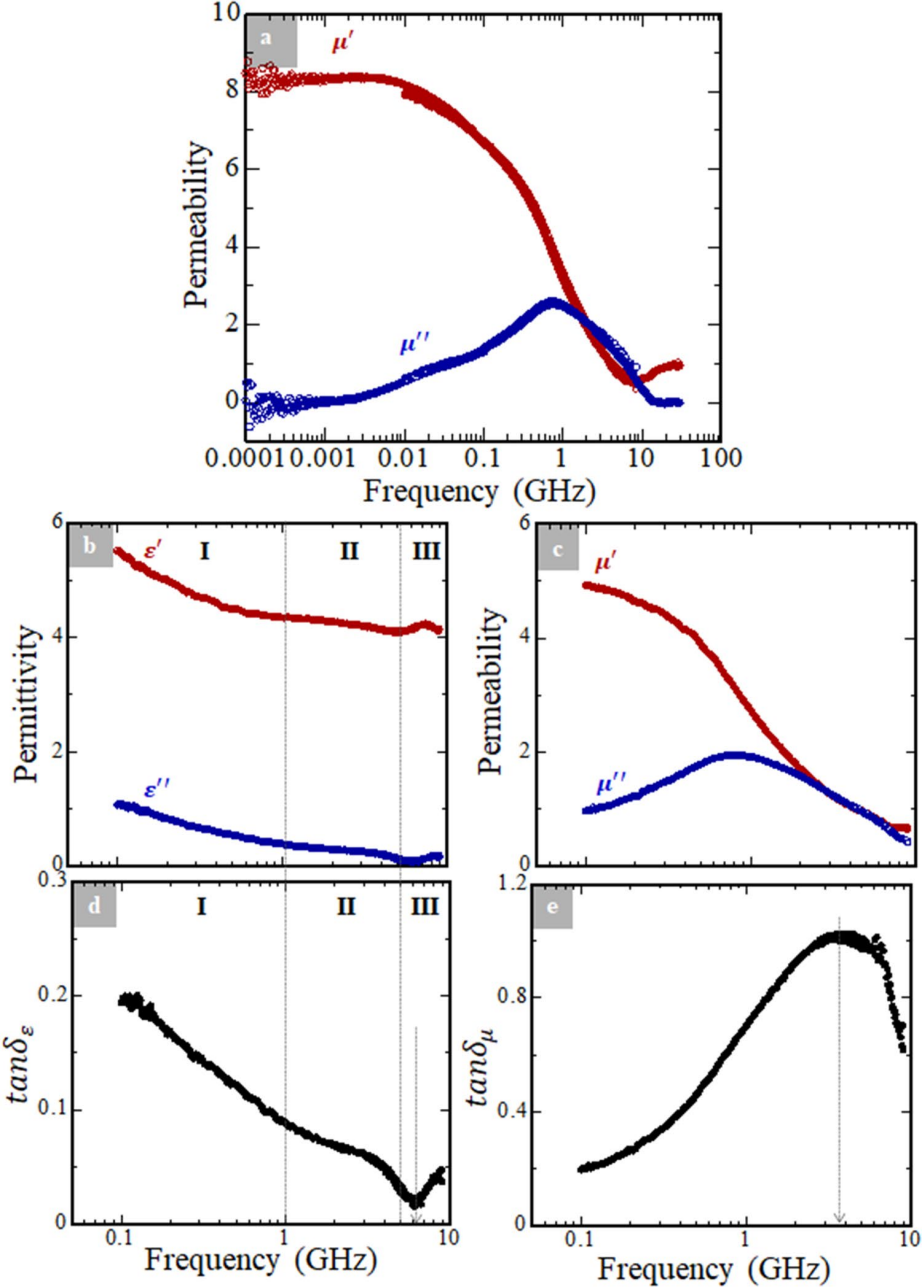
(**a**) Frequency dependence of dynamic permeability evaluated through the short-circuited MSL method. The maximum frequency of irradiated electromagnetic wave is 30 GHz. Frequency dependences of (**b**) the complex permittivity and (**c**) the complex permeability measured by means of a network analyzer in the frequency range of 0.1–9 GHz. For these measurements, the present Ni_0.5_Zn_0.5_Fe_2_O_4_ powder/polymer composite sheet was fabricated as a toroidal shape. (**d**) The ${\text{tan}}\delta_{\varepsilon } = \frac{{\varepsilon ^{\prime \prime }}}{{\varepsilon {\prime }}}$, and (**e**) ${\text{tan}}\delta_{\mu } = \frac{{\mu ^{\prime \prime }}}{{\mu {\prime }}}$, respectively, calculated using the results obtained from (**b**) and (**c**), respectively.

The complex permeability of the Ni_0.5_Zn_0.5_Fe_2_O_4_-powder/polymer composite sheet measured by the short-circuited MSL method^10,15,19^ is shown in Fig. 3a where the real ($\mu ^{\prime }$) and imaginary ($\mu^ {\prime \prime }$) parts of the relative permeability are plotted against the frequency of electromagnetic wave irradiated up to 30 GHz. When increasing the frequency, a gradual decrease was observed in the $\mu^ {\prime } - f$ profile, whereas a peak was observed in the $\mu ^{\prime \prime } - f$ profile. We define $f_{r}$ as the frequency at the maximum position of the $\mu ^{\prime \prime } - f$ curve ($\mu ^{\prime \prime }_{max}$). From Fig. 3a, $f_{r}$ at $\mu ^{\prime \prime }_{max}$ is ca. $7.5 \times 10^{ - 1}$ GHz. At this frequency, the relative $\mu^{\prime }$ and $\mu ^{\prime \prime }$ values of the present sample are 3.4 and 2.6, respectively. For a 0.3(Li_2_MoO_4_)–0.7(Ni_0.5_Zn_0.5_Fe_2_O_4_) sample mixed with PVA, with a composition similar to the present sheet, the following values were reported: 4.0 ($\mu^{\prime }$) and 1.0 ($\mu ^{\prime \prime }$)^24^. Our sample showed 6.8 for $\mu^{\prime }$ and 1.4 for $\mu ^{\prime \prime }$ at 0.1 GHz, whereas the 0.3(Li_2_MoO_4_)–0.7(Ni_0.5_Zn_0.5_Fe_2_O_4_) composite exhibited 8.8 ($\mu ^{\prime }$) and 0.2 ($\mu ^{\prime \prime }$) at the same frequency. Dynamic permeability of the present sheet would be regarded as comparable to that of the 0.3(Li_2_MoO_4_)–0.7(Ni_0.5_Zn_0.5_Fe_2_O_4_) composite, measured using a different measurement system and method^24^. This confirms the reliability of the present evaluation method.

The high-frequency electromagnetic wave loss phenomena should be addressed. The frequency dependence of the complex permittivity (Fig. 3b) and complex permeability (Fig. 3c) were measured using a network analyzer (ENA E5080A, Keysight Technologies Inc.) on the present sheet sample fabricated in a toroidal shape. We measured in the frequency range of 0.1 to 9 GHz in these experiments. From the obtained permittivity and permeability, the loss tangents, i.e., ${\text{tan}}\,\delta_{\varepsilon } = \frac{{\varepsilon^{\prime\prime}}}{{\varepsilon {\prime }}}$ (Fig. 3d) and ${\text{tan}}\,\delta_{\mu } = \frac{{\mu^{\prime\prime}}}{{\mu {\prime }}}$ (Fig. 3e), respectively, were calculated.

We inferred that the permittivity (Fig. 3b) and ${\text{tan}}\,\delta_{\varepsilon }$ (Fig. 3d) can be categorized into three regions, I, II, and III. In the region I, the low frequency range of $f \le 1\,{\text{GHz}}$, the dispersion of permittivity (Fig. 3b) and ${\text{tan}}\,\delta_{\varepsilon }$ (Fig. 3d) rapidly decreased. According to Patil et al.^25^, the decrease in permittivity at low frequencies is attributed to the interfacial polarization between two different phases; Ni_0.5_Zn_0.5_Fe_2_O_4_ and PVA in the present case. Differing permittivies and conductivities between Ni_0.5_Zn_0.5_Fe_2_O_4_ and PVA induce space charges under the application of an electric field on the composite sheet. The space charges accumulate at the interface between Ni_0.5_Zn_0.5_Fe_2_O_4_ and PVA, leading to interfacial polarization^25,26^.

The response time of the interfacial polarization is denoted as^25^,

1 $$\tau = \frac{1}{{\omega_{max} }},$$

where $\omega_{max} = 2\pi f_{max}$. In the low frequency region, which corresponds to region I in this case, $\tau$ is relatively long^25^ according to Eq. (1). Consequently, the decrease in permittivity is prominent in region I. This is the reason for the relatively dull-negative slopes of permittivity (Fig. 3b) and ${\text{tan}}\,\delta_{\varepsilon }$ (Fig. 3d) in the range of $1 < f \le 5\,{\text{GHz}}$ (region II), compared with region I.

On the other hand, region III is highly arguable. We observed an anomalous peak in $\varepsilon^{\prime } - f$ profile at $\sim 6.1\,{\text{GHz}}$ (Fig. 3b). The plausible explanation for region III is electron hopping. We previously described that our sample exhibited a high surface resistivity value. However, it does not mean that electrons responsible for conduction of the sample do not exist. Namely, an electron hopping between ${\text{Fe}}^{2 + } /{\text{Fe}}^{3 + }$ and between ${\text{Ni}}^{3 + } /{\text{Ni}}^{2 + }$ could partially account for the conduction^25^, though the conductivity is quite low in our sample. Note that the Mössbauer measurements concluded that the present Ni_0.5_Zn_0.5_Fe_2_O_4_ ceramic powder exhibited ${\text{Fe}}^{3 + }$ only, but the detection limit of the Mössbauer measurements is approximately 2% of Fe. Thus, small amounts of ${\text{Fe}}^{2 + }$ could exist in the present sample. When the sample is subjected to high-frequency electromagnetic waves, the electron hopping between ${\text{Fe}}^{2 + } /{\text{Fe}}^{3 + }$ and between ${\text{Ni}}^{3 + } /{\text{Ni}}^{2 + }$ cannot be neglected; the electron hopping between these cation pairs leads to a local displacement of the space charges, resulting in the interfacial polarization in accordance with the direction of the electric field^25,27,28^. When the frequency of irradiated-electromagnetic waves on the sample is increased in region III, the electron hopping would be activated and frequent. Consequently, the interfacial polarization of the sample tends to be increased. After passing through the “critical frequency” at $\sim 6.1\,{\text{GHz}}$, the electron hopping cannot follow the alternating electric field any more^26^, which leads to an abrupt deterioration of permittivity (Fig. 3b). This could be the reason for the anomalous peak^25^ in Fig. 3b, while a minimum value was observed in Fig. 3d owing to an inverse relationship between $\varepsilon^{\prime }$ and ${\text{tan}}\,\delta_{\varepsilon }$. We speculate that the anomalous peak in $\varepsilon^{\prime } - f$ profile in category III (Fig. 3b) is due to the electron hopping.

We further measured frequency dependence of permeability (Fig. 3c) to discuss the magnetic loss, ${\text{tan}}\delta_{\mu }$ (Fig. 3e). We obtained the maximum ${\text{tan}}\delta_{\mu }$ value of approximately 1.0 at $\sim 3.6\,{\text{GHz}}$ (Fig. 3e). As is generally recognized, ${\text{tan}}\delta_{\mu }$ of magnetic materials consists of the sum of eddy-current loss (${\text{tan}}\delta_{e}$), hysteresis loss (${\text{tan}}\delta_{h}$) and remanent loss (${\text{tan}}\delta_{r}$). As previously mentioned, the influence of ${\text{tan}}\delta_{e}$ is negligible for the present sheet. Thus, we consider only ${\text{tan}}\delta_{h}$ and ${\text{tan}}\delta_{r}$.

To discuss ${\text{tan}}\delta_{h}$, we must consider the stress sensitivity of the samples. In general, stresses such as magnetostriction, compressive and tensile stresses of magnetic materials cause stress-induced deformation of the hysteresis loops, which give rise to a non-zero ${\text{tan}}\delta_{h}$^29,30^ in the high-frequency band. For polycrystalline samples, the stress effects and hence also ${\text{tan}}\delta_{h}$ are complicated because various types of stresses are possible with random directions^31^. When we prepared the Ni_0.5_Zn_0.5_Fe_2_O_4_-powder/polymer composite sheet, we ground the Ni_0.5_Zn_0.5_Fe_2_O_4_ ceramic powder sufficiently to attain fine Ni_0.5_Zn_0.5_Fe_2_O_4_ particles. It is possible that this procedure could generate some internal stresses on the Ni_0.5_Zn_0.5_Fe_2_O_4_ particles. On the other hand, mechanical or electric stresses were not applied for the present sample, so that the present sheet should be free from external stresses. ${\text{tan}}\delta_{h}$ consists of the sum of internal and external stresses, but the areas of the hysteresis loops of the sheet (Fig. 2e) are small. Hence, an influence of ${\text{tan}}\delta_{h}$ is rather small for the present case.

For ${\text{tan}}\delta_{r}$, the resonance linewidths of the $\mu^{\prime } - f$ and $\mu ^{\prime \prime } - f$ profiles are good indicators, because the linewidths determine the amount of ${\text{tan}}\delta_{r}$^31^. It has been reported that four independent factors determine the resonance linewidths, and hence ${\text{tan}}\delta_{r}$: (1) spin–lattice relaxation, (2) porosity and/or nonmagnetic inclusions, (3) magnetocrystalline anisotropy, and (4) surface roughness. (2) plays the role of local demagnetization centers to induce demagnetization effects owing to inhomogeneous internal magnetic fields. This results in broadening of the resonance linewidths^32,33^. We infer that (2) would be the most probable origin of ${\text{tan}}\delta_{r}$ for the present composite material among these four factors.

As a conclusion, the ${\text{tan}}\delta_{\mu }$ value in the present Ni_0.5_Zn_0.5_Fe_2_O_4_-powder/polymer composite sheet (Fig. 3e) could be mainly due to ${\text{tan}}\delta_{r}$, which is caused by (2). In this study, we achieved dynamic-physical behavior for our Ni_0.5_Zn_0.5_Fe_2_O_4_-powder/polymer composite sheet comparable to that previously reported for a material with essentially similar composition^24^, except the ${\text{tan}}\delta_{\mu }$ value of ~ 1.0 in the vicinity of $\sim 3.6\,{\text{GHz}}$ (Fig. 3e). To overcome large loss tangents, the formation of epitaxially-grown single-crystal Ni_0.5_Zn_0.5_Fe_2_O_4_ films on the flexible underlayers could be a solution.

**Figure 4:**
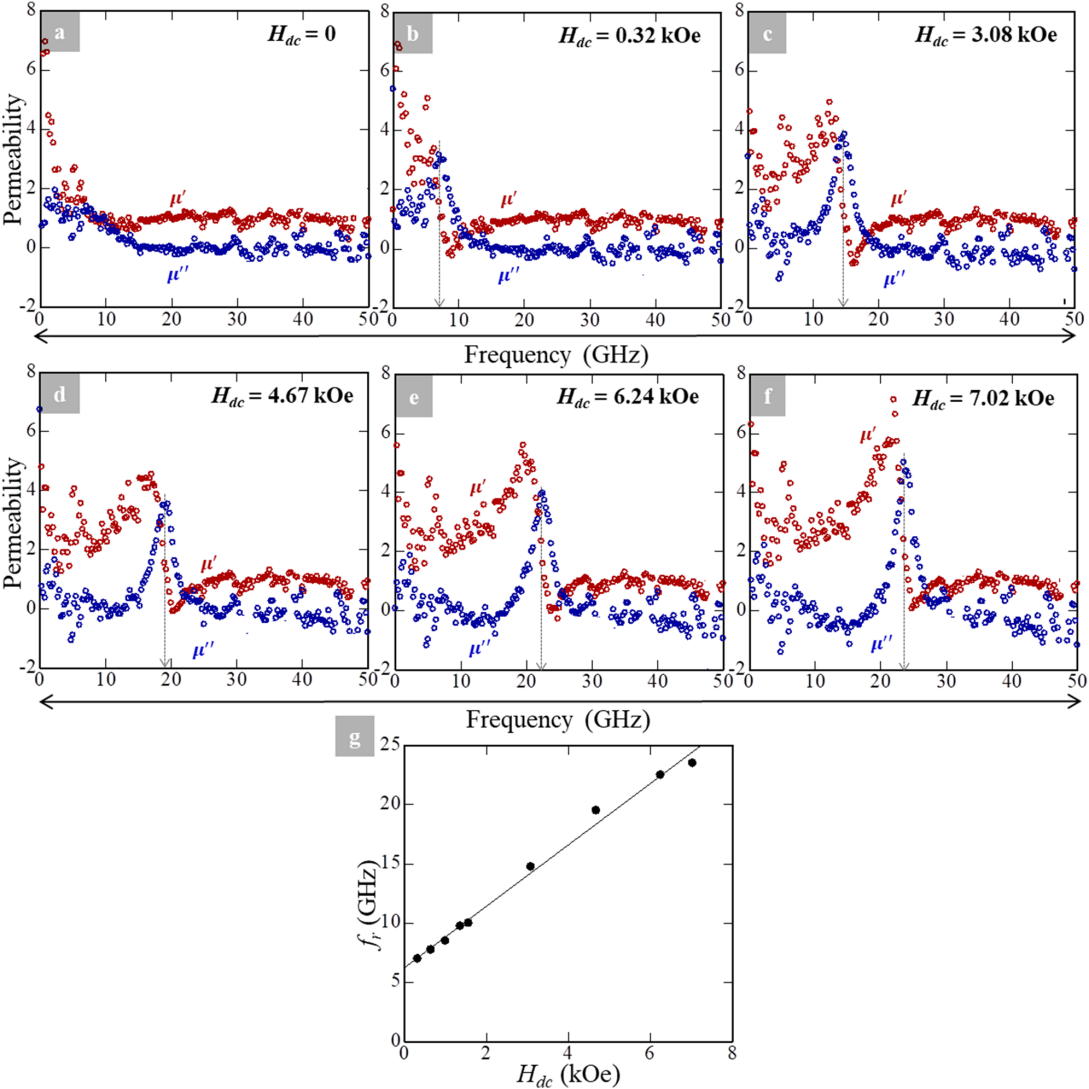
Frequency dependence of dynamic permeability obtained by the MSL-probe method up to 50 GHz. The external magnetic DC field ($H_{dc}$) applied during the measurements is (**a**) 0, (**b**) 0.32, (**c**) 3.08, (**d**) 4.67, (**e**) 6.24, and (**f**) 7.02 kOe, respectively. (**g**) The relationship between $f_{r}$ and $H_{dc}$ based on the results form measurements with a linear fit.

We also evaluated the complex permeability by the MSL-probe method, (ii). Although the methods (i) and (ii) represent different approaches (see Method section), we emphasize that the dynamic $\mu^{\prime } - f$ and $\mu ^{\prime \prime } - f$ behaviors were essentially consistent. In the MSL-probe method, we applied a strong static external magnetic field, $H_{dc}$, on the sheet sample during the measurements. The strength of $H_{dc}$ was controlled by changing the strength of an applied DC current with/without using yokes. As a consequence, $H_{dc}$ was varied from 0 to ~ 7 kOe. In this case, $H_{dc}$ was applied parallel to a high frequency-alternating current (AC), $I_{rf}$, which propagates along the MSL. As a result, the configuration of the directions among longitudinal direction of the rectangle-shaped sheet sample, $H_{dc}$, and $I_{rf}$ are parallel. Figure 4a–f show the results of the $\mu^{\prime } - f$ and $\mu ^{\prime \prime } - f$ behaviors upon applying various $H_{dc}$ fields obtained by method (ii). In contrast to Fig. 3a, these figures are plotted with a linear scale for the X-axis. The maximum frequency value of electromagnetic wave irradiated for these measurements is 50 GHz.

Note that the measurement condition of Fig. 3a is the same as that of Fig. 4a ($H_{dc} = 0$). Upon increasing $H_{dc}$, the position of $\mu ^{\prime \prime }_{max}$ (indicated by dashed arrows) shifted towards higher frequencies. Changes in $f_{r}$ as a function of $H_{dc}$ are summarized in Fig. 4g; we clarified the apparent shift of FMR through $H_{dc}$. From Fig. 4g, the relationship between $f_{r}$ and $H_{dc}$ assuming a linear relation is:

2 $$f_{{r\left( {cal} \right)}} = 2.59H_{dc} + 6.25,$$

with a reliability value, $\left| r \right|$, of $9.96 \times 10^{ - 1}$. The linear relationship between $f_{{r\left( {cal} \right)}}$ and $H_{dc}$ in Fig. 4g is reasonable from the perspective of the FMR theory reported by Kittel^34^. In the present case, the linearity is concluded for the entire range of $H_{dc}$ (Fig. 4g), such that Ni_0.5_Zn_0.5_Fe_2_O_4_-powder/polymer composite sheet could be applicable in a wide range of GHz-operational frequencies^35^ even at small $H_{dc}$.

From Eq. (2), the value of $f_{{r\left( {cal} \right)}}$ is determined to be 6.25 GHz at $H_{dc} = 0$. However, we did not observe any apparent $\mu ^{\prime \prime }_{max}$ in Fig. 4a ($H_{dc} = 0$), and hence the determination of $f_{r}$ is impossible from Fig. 4a. This is contradictory to the experiment (Fig. 4a) and the mathematical extrapolation [Eq. (2)]. We should also mention that the $f_{r}$ value is estimated to be $7.5 \times 10^{ - 1}$ GHz from Fig. 3a ($H_{dc} = 0$); this is the only difference between methods (i) and (ii), which reveals the difficulties of precise evaluations under GHz-frequency bands.

**Figure 5:**
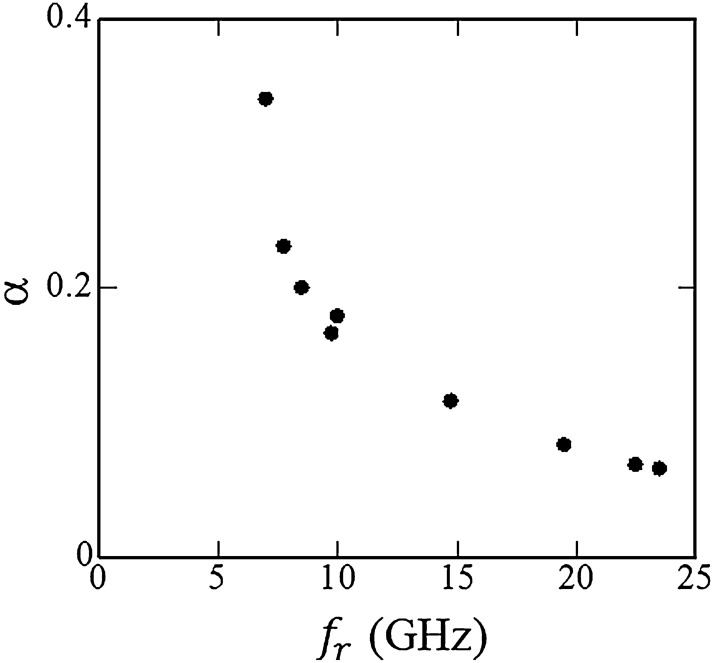
Gilbert damping constant, $\alpha$, as a function of the resonance frequency ($f_{r}$). $f_{r}$ values are obtained from Fig. 4g. On the other hand, α values are determined by the $\mu ^{\prime \prime } - f$ profiles from method (ii) using Eq. (3).

We studied a relationship between the Gilbert damping constant, α, and $f_{r}$ (Fig. 5). For this discussion, $f_{r}$ values were obtained from Fig. 4g, whereas α values were determined by the $\mu ^{\prime \prime } - f$ profiles obtained by method (ii). We utilized following equation^36^ for calculating the damping constant,

3 $$\upalpha = \frac{\Delta f}{{f_{r} }}.$$

$\Delta f$ is denoted as $\frac{1}{2} \times$ (full width at half maximum of the $\mu ^{\prime \prime } - f$ peak).

From Fig. 5, we found that the damping constant drastically decreased as $f_{r}$ increases (increases in $H_{dc}$). The $\alpha_{max}$ value is $3.40 \times 10^{ - 1}$ at $f_{r} = 7.01$ GHz ($H_{dc} = 3.20 \times 10^{ - 1}$ kOe, which corresponds to Fig. 4b), while the value of $\alpha_{min}$ is 6.52 $\times 10^{ - 2}$ at $f_{r} = 2.35 \times 10$ GHz ($H_{dc} = 7.02$ kOe corresponding to Fig. 4f). In epitaxially-grown spinel ferrite films with the chemical formula of Ni_0.65_Zn_0.35_Al_0.8_Fe_1.2_O_4_ possessing a similar composition as the present sample, low damping constant values have been reported; $2.6 \times 10^{ - 3} \le \alpha \le 3.5 \times 10^{ - 3}$^37^. These values are one-to-two orders of magnitude lower than our sample.

A magnetic-based material exhibiting a high $\upalpha$ value implies that the FMR phenomenon of the sample is complicated^38^. In the case of spinel-structured ferrite films, the constituent of cations^39^ and high density of crystal defects generated by dislocations and/or antiphase boundaries^40−42^ affect the damping. This results in a defect-mediated damping^37^. These factors are potentials for “extrinsic” damping, which leads to high $\alpha$ value^43−48^. However, from microstructural investigations in this study, we observed neither remarkable antiphase boundaries nor misfit dislocations (Fig. 1c–e). In the magnetic field dependent magnetization behaviors, the structural defects such as antiphase boundaries and so on could lead to a large $H_{c}$ value and “S-shaped” hysteresis curve^49^, but we obtained a rather small value for $H_{c}$ as well as “regular-shaped” hysteresis curves (Figs. 1f and 2e). By considering these experimental facts, we can eliminate the possibility of defect-mediated damping behind the high $\alpha$ values in the present case.

By taking into account the discussions above, we suggest that the damping of the present Ni_0.5_Zn_0.5_Fe_2_O_4_ powder/polymer composite sheet could be largely related to the 3*d* electrons from Fe and Ni atoms. Although further detailed studies are necessary, intuitive considerations would be possible; the coupling between 3*d* electrons, 3*d* spins, and phonons is sufficiently strong to induce high $\alpha$ values^38^ in the present Ni_0.5_Zn_0.5_Fe_2_O_4_ powder/polymer composite sheet. In the epitaxially-grown spinel structured MgFe_2_O_4_ thin film deposited on MgAl_2_O_4_ (100) substrate, the $\alpha$ value was reported to be approximately $8 \times 10^{ - 2}$^49^. Our $\alpha_{min}$ value ($6.52 \times 10^{ - 2}$ at $f_{r} = 2.35 \times 10$ GHz with the application $H_{dc}$ of 7.02 kOe) is comparable to that value^49^, though the present sheet consists of polycrystalline Ni_0.5_Zn_0.5_Fe_2_O_4_ particles.

Therefore, the significance of the present study is the high potential of the present Ni_0.5_Zn_0.5_Fe_2_O_4_ powder/polymer composite sheet composed of an environmentally friendly ferrite with cost-effective elements applicable over GHz bands. Another prominent significance of this study is showing the reliability of the novel evaluation methods (i) and (ii). This study gives hope for the establishment of high-frequency technologies beyond several tens of GHz bands.

## Conclusions

In conclusion, we applied two different originally developed techniques, short-circuited MSL^10,15,19^ and MSL-probe^11,20^ methods, to a Ni_0.5_Zn_0.5_Fe_2_O_4_ powder/polymer composite sheet prepared from high-quality Ni_0.5_Zn_0.5_Fe_2_O_4_ ceramic powder. Although the two techniques are based on different experimental setups/systems, the dynamic permeability behaviors at GHz bands are essentially consistent.

A linear relationship between $f_{{r\left( {cal} \right)}}$ and $H_{dc}$ was obtained for the entire measured range, up to 50 GHz. The linearity between $f_{{r\left( {cal} \right)}}$ and $H_{dc}$ at sufficiently small required $H_{dc}$ suggests that the present sheet has potential applications to power-efficient devices^37^. Further study on the damping is necessary, but the damping process of the current Ni_0.5_Zn_0.5_Fe_2_O_4_ powder/polymer composite sheet could be dominated by a strong coupling between 3*d* electrons, 3*d* spins, and phonons.

Although the present sheet is composed of polycrystalline Ni_0.5_Zn_0.5_Fe_2_O_4_, the sheet showed soft magnetism exhibiting parallel magnetic anisotropy, high surface resistivity, small required $H_{dc}$ for FMR, an intrinsic damping process, and comparable damping constant to the epitaxially-grown thin film^49^. These results indicate that high-performance devices that can be utilized in frequency bands up to several tens of GHz. For example, the present Ni_0.5_Zn_0.5_Fe_2_O_4_-powder/polymer composite sheet is magnetically anisotropy, such that “anisotropic suppression” of electromagnetic waves with GHz frequencies could be possible^50^; the present sample can be expected to be useful as an anisotropic-EMI material in the GHz band, which is essential for high-frequency electronic devices.

## Methods

### Synthesis of impurity-free spinel-structured Ni_0.5_Zn_0.5_Fe_2_O_4_ powder

In the present study, Ni_0.5_Zn_0.5_Fe_2_O_4_ powder was mixed with a polymer to attain a Ni_0.5_Zn_0.5_Fe_2_O_4_ powder/polymer composite sheet. First of all, Ni_0.5_Zn_0.5_Fe_2_O_4_ ceramic powder was synthesized by a solid-state reaction method. High purity starting materials, i.e., stoichiometric quantities of α-Fe_2_O_3_ (99.9%), NiO (99.97%), and ZnO (99.999%), were mixed and calcined in air at 1000 °C for 4 h. The calcined powder was ground and pressed to pellets with the diameter of 10 mm, by applying a mechanical pressure of 10 MPa. Finally, the pelletized sample was annealed in air at 1400 °C for 4 h. The annealing temperature was chosen to achieve the most superior sample quality. To avoid peroxidation, we annealed the sample in air instead of an O_2_ atmosphere.

### Preparation of Ni_0.5_Zn_0.5_Fe_2_O_4_ powder/polymer composite sheet by the Doctor–Blade method

The pelletized Ni_0.5_Zn_0.5_Fe_2_O_4_ after the final annealing was ground carefully using a grinder to obtain Ni_0.5_Zn_0.5_Fe_2_O_4_ particles with a homogeneous size-distribution. We classified the Ni_0.5_Zn_0.5_Fe_2_O_4_ particles using sieves with different mesh sizes. After that, the Ni_0.5_Zn_0.5_Fe_2_O_4_ powder with homogeneous size was mixed with a PVA (–(CH_2_CH(OH)–)_500_) solution. The ceramic Ni_0.5_Zn_0.5_Fe_2_O_4_ powder and PVA solution were well mixed in a crucible, resulting in slurry with proper viscosity. By spreading the slurry on a flat PET-film and applying the Doctor-Blade method, we obtained sheet-shaped Ni_0.5_Zn_0.5_Fe_2_O_4_ powder/polymer composite. After drying the sheet under ambient conditions in air, the sheet was removed from PET film, and was heat-treated to dry completely at 105 °C for 1 h; this is a monolayered sheet with the thickness of 166 μm on the average. Afterward, we piled two monolayered sheets, and performed a heat treatment at 150 °C for 6 min by applying a mechanical pressure to attain higher density of the Ni_0.5_Zn_0.5_Fe_2_O_4_ powder/polymer composite sheet. We focus only on the double-layered Ni_0.5_Zn_0.5_Fe_2_O_4_ powder/polymer composite sheet instead of a monolayered one in this study.

### Fundamental characterizations

The phase purity and crystallinity were evaluated by X-ray diffraction (XRD, SmartLab, Rigaku Corporation). The magnetic field dependent magnetization behaviors were measured in the range of ± 10 kOe at room temperature using a vibrating sample magnetometer (VSM, C7-10A, Toei Industry Co., Ltd.). The Ni_0.5_Zn_0.5_Fe_2_O_4_ powder was embedded in a polymer matrix (Pattex 100% glue, Henkel) on a Fe-free Al foil, and room-temperature ^57^Fe Mössbauer spectra were collected at 12 mm/s (FGX-222ST Mössbauer spectrometer, Topologic Systems Inc.) using a 50 mCi Cyclotron ^57^Co/Rh source. The microscopic crystal structures of the sample were observed by field-emission scanning electron microscopy (FE-SEM, JSM-7000F, JEOL Ltd.) and field-emission transmission electron microscopy (FE-TEM, JEM-ARM200F, JEOL Ltd.), respectively.

### Evaluations of dynamic permeability in the GHz-frequency region

For the dynamic permeability in the GHz-frequency region, (i) short-circuited MSL^10,15,19^ and (ii) MSL-probe^11,20^ methods were employed and their results were compared. In the case of (i), originally developed sample jigs were used to produce electromagnetic radiation of suitable frequencies to irradiate the sample for the measurements. The sheet sample was cut into a rectangle and placed at the short end of the MSL. In method (i), a sufficiently strong $H_{dc}$ was applied to saturate the Ni_0.5_Zn_0.5_Fe_2_O_4_ powder/polymer composite sheet using a permanent magnet (Fig. 3a). $I_{rf}$, AC with high frequency, was propagated along the MSL, such that an AC magnetic field, $H_{rf}$, is generated perpendicular to the direction of the $I_{rf}$. In this method, we regarded the overall experimental system as an “electric circuit model” including the sample and the jigs^10,15,19^. We attained one of the scattering matrixes of an electric circuit, $S_{11}$ as an output signal from the measurements in this method. Using $S_{11}$, the admittance,$Y_{1}$, of the electric circuit can be calculated. $H_{dc}$ was applied parallel to the longitudinal direction of the rectangle-shaped sheet sample. Therefore, the direction of $H_{dc}$ is perpendicular to $I_{rf}$. Under the application of $H_{dc}$, the electric circuit model of the experimental system changes, so that $Y_{1}$ is replaced by $Y_{2}$^10,15,19^. By the use of $Y_{1}$ and $Y_{2}$, we can determine the relative values of complex permeability of the sample. The details of the method and calculation procedures are mentioned elsewhere^10,15,19^.

In method (ii), a “self-developed” probe composed of a MSL on a flexible substrate was used for the measurements^11,20^. The MSL probe was set on the surface of sheet sample with a gap between the sample and the probe of approximately 200 μm. Before the measurements, a calibration of the network analyzer had been carefully performed by applying $H_{dc} \approx 8 {\text{kOe}}$ without the sample. Non-magnetic signals were perfectly subtracted from coaxial cables and jigs throughout the calibration. Similar to method (i), we applied sufficiently strong $H_{dc}$ after the sheet sample was placed in the measurement device in the method (ii). This procedure enables us to saturate the specimen^20^. After that, the permeability measurements were carried out. In this method, the overall experimental system is also regarded as an electric circuit model. The output signal from the measurements is $S_{21}$, which is one of the other scattering matrixes of an electric circuit. Using the obtained $S_{21}$, we can determine the relative values of complex permeability of the sample. For the details of method (ii), refer to literatures^11,20^.

## Data Availability

All data generated or analyzed during this study are included in this published article.
